# The Mini-Addenbrooke's Cognitive Examination: A New Assessment Tool for Dementia

**DOI:** 10.1159/000366040

**Published:** 2014-09-11

**Authors:** Sharpley Hsieh, Sarah McGrory, Felicity Leslie, Kate Dawson, Samrah Ahmed, Chris R. Butler, James B. Rowe, Eneida Mioshi, John R. Hodges

**Affiliations:** ^a^Brain and Mind Research Institute, University of New South Wales, Sydney, N.S.W., Australia; ^b^Neuroscience Research Australia, University of New South Wales, Sydney, N.S.W., Australia; ^c^ARC Centre of Excellence in Cognition and Its Disorders, University of New South Wales, Sydney, N.S.W., Australia; ^d^School of Medical Sciences, University of New South Wales, Sydney, N.S.W., Australia; ^e^Alzheimer Scotland Dementia Research Centre, Department of Psychology, University of Edinburgh, Edinburgh, UK; ^f^Departments of Clinical Neurosciences, University of Oxford, John Radcliffe Hospital, Oxford, UK; ^g^Psychiatry, Cambridge University, University of Oxford, John Radcliffe Hospital, Oxford, UK; ^h^Behavioural and Clinical Neuroscience Institute, University of Oxford, John Radcliffe Hospital, Oxford, UK; ^i^Medical Research Council, Cognition and Brain Sciences Unit, Cambridge, University of Oxford, John Radcliffe Hospital, Oxford, UK; ^j^Nuffield Department of Clinical Neurosciences, University of Oxford, John Radcliffe Hospital, Oxford, UK

**Keywords:** Cognitive screening test, Frontotemporal dementia, Alzheimer's disease, Corticobasal degeneration

## Abstract

**Background/Aims:**

We developed and validated the Mini-Addenbrooke's Cognitive Examination (M-ACE) in dementia patients. Comparisons were also made with the Mini Mental State Examination (MMSE).

**Method:**

The M-ACE was developed using Mokken scaling analysis in 117 dementia patients [behavioural variant frontotemporal dementia (bvFTD), n = 25; primary progressive aphasia (PPA), n = 49; Alzheimer's disease (AD), n = 34; corticobasal syndrome (CBS), n = 9] and validated in an independent sample of 164 dementia patients (bvFTD, n = 23; PPA, n = 82; AD, n = 38; CBS, n = 21) and 78 controls, who also completed the MMSE.

**Results:**

The M-ACE consists of 5 items with a maximum score of 30. Two cut-offs were identified: (1) ≤25/30 has both high sensitivity and specificity, and (2) ≤21/30 is almost certainly a score to have come from a dementia patient regardless of the clinical setting. The M-ACE is more sensitive than the MMSE and is less likely to have ceiling effects.

**Conclusion:**

The M-ACE is a brief and sensitive cognitive screening tool for dementia. Two cut-offs (25 or 21) are recommended.

## Introduction

The assessment of cognition is critical for the detection and differential diagnosis of dementias [1]. It has important clinical implications for patient care, including access to treatment options, indicators for rates of survival, competency to drive and capacity to give informed consent, plus psychosocial factors such as whether patients are able to live independently at home, carer burden and quality of life. Shorter cognitive screening tests are particularly useful in a busy clinic where resources may not permit more comprehensive neuropsychological testing. The Addenbrooke's Cognitive Examination-III (ACE-III) and its predecessor, the ACE-R, are widely adopted and validated tools currently used in memory clinics and dementia research around the world [2,3,4,5,6]. They are useful in the detection and differentiation of dementia subtypes [7,8]. The ACE-III is the latest version of the ACE, which has been validated against formal neuropsychological tests [3]. It is scored out of 100 and includes items assessing the domains of attention, memory, fluency, language and visuospatial function. However, it is a test that requires at least 15-20 min to administer which is beyond the scope of many clinical settings. The aim of this study was to derive a shorter version of the ACE-III, the Mini-Addenbrooke's Cognitive Examination (M-ACE), using a data-driven scaling method, to validate this new instrument in patients with a range of dementia syndromes and to compare the M-ACE with the widely used commercially available Mini Mental State Examination (MMSE) [9].

## Methods

### Participants

Participants were recruited from the Frontier Research Group, Sydney, N.S.W., Australia, the Memory Clinic of Cambridge University Hospitals NHS Trust, Cambridge, United Kingdom and the Oxford Cognitive Disorders Clinic, Oxford, United Kingdom. Patients were assessed in multidisciplinary teams including an experienced behavioural neurologist, and diagnosis was made in line with current diagnostic criteria [10,11,12,13] according to the clinical assessment, comprehensive neuropsychological assessment and structural brain imaging. Controls were recruited from a healthy volunteer panel. Exclusion criteria for controls were the presence of significant neurological conditions (e.g., epilepsy), current psychiatric illness (e.g., depression) and significant drug and alcohol history.

### Standard Protocol Approvals, Registrations, and Patient Consents

All participants provided informed consent for the study. Where necessary, the carer for some patients provided consent. The study was approved by the Southern Sydney Illawarra Area Health Service and University of New South Wales ethics committees in Sydney, the Cambridge II Research Ethics Committee and the Oxfordshire Research Ethics Committee.

### Derivation of the M-ACE

Mokken scaling analysis [14] was used to examine the hierarchy of items on the ACE-III and derive the M-ACE. This technique provides an indication of test item difficulty and discriminatory ability. Ideally, a sound instrument consists of items covering a wide range of difficulty levels with high discrimination. Briefly, Mokken scaling analysis first seeks unidimensional sets of items based on several scalability coefficients first introduced by Loevinger [15]. For the entire set of items, there is a test scalability coefficient (H), for each item within a test there is an item scalability coefficient (H_i_) and for each item pair an item pair scalability coefficient (H_ij_). H is a measure of the extent to which pairs of test items completed by the participant appear in the same relative order which ranges from 0 (no scalability) to 1 (perfect ordering) and 0.3 is generally taken as minimum value for a Mokken scale. A set of items form a Mokken scale provided all item scalability coefficients are greater than 0.3 and for all item pairs, the item pair scalability coefficient is a positive value [16]. Next, Mokken scaling analysis identifies items that conform to the monotone homogeneity model (MHM). Scores on items conforming to this model increase as the level of the latent trait (e.g., cognitive ability) increases and items that do not meet the MHM criteria can be removed. Once items fit the MHM, H_i_ can be interpreted as a measure of the items discrimination with higher values indicating greater discrimination [17]. Finally, unidimensional sets of items meeting the MHM criteria can be examined for invariant item ordering (IIO), which is necessary in the development of hierarchies that are replicable across samples. Invariantly ordered items are also responded to in the same order by all respondents regardless of the participant's level of cognitive ability. IIO identifies items with item response functions that do not overlap. The H-trans (H^T^) refers to the distance between the item response functions with higher values indicating greater IIO accuracy [18].

Itemized ACE-III data from 117 patients [behavioural variant frontotemporal dementia (bvFTD), n = 25; primary progressive aphasia (PPA), n = 49; Alzheimer's disease (AD), n = 34; corticobasal syndrome (CBS), n = 9; mean age = 65.5 ± 8.2 years; mean years of education = 12.4 ± 3.1] were used for Mokken scaling analysis. The mean score of each ACE-III item was divided by the maximum number of points available for that item to equate scores across all of the items for comparison purposes [e.g., a mean score of 3.97 for ‘orientation to time’ was divided by the maximum score possible for that item (5) to produce an equated mean of 0.74]. See online supplementary table 1 (see www.karger.com/doi/10.1159/000366040 for all online suppl. material) for the complete list of equated means in the patient cohort. ‘Naming’, which is scored from 0 to 12, was rescaled to 0-9 as this method does not accommodate values above 9. Data were entered into the freeware R environment [19] using the ‘Mokken’ package [20].

First, examining the unidimensionality of the data showed that all item pair scalability coefficients (H_ij_) were positive. The H_i_ values of 5 of the 24 ACE-III items fell below the 0.3 threshold level (‘repetition of single multisyllabic words’, ‘perceptual dots’, ‘overlapping infinity loops’, ‘single word reading’, ‘drawing of a cube’) and were excluded. The remaining 19 items yielded a combined H of 0.45. Second, no items were excluded in the assessment of monotonicity. Finally, in the assessment of IIO, 2 items (‘syntactical comprehension’ and ‘3-item registration’) were removed. The remaining 17 items formed a moderate hierarchical Mokken scale (H = 0.44) with IIO (H^T^ = 0.61) (see table 2 in online suppl. material). From these 17 items, 5 items were selected based on a conceptually driven decision to cover the main cognitive domains. These include: orientation to time, learning and recall of the name and address, animal fluency and drawing of a clock face to command with time specification (ten past five o'clock). The selected items range in difficulty where equated means scores ranged from 0.33 (most difficult) to 0.78 (least difficult) (see table 2 in online suppl. material). The items also have high discrimination (H_i_ = 0.42-0.52). Within the orientation items, ‘season’ was removed as this particular question is known to be difficult to answer in some regions (e.g., tropics). These 5 items together form the M-ACE, which has a maximum score of 30 with higher scores indicating better cognitive function.

### Data Analyses for Validation of the M-ACE and Comparisons with the MMSE

Data were analysed using IBM SPSS (Version 20). Non-parametric tests were carried out as data were non-normally distributed. In all analyses, a conservative threshold of p < 0.01 was used. An independent sample of 242 participants consisting of 164 dementia patients (bvFTD, n = 23; PPA, n = 82; AD, n = 38; CBS, n = 21) and 78 controls was used to validate the M-ACE. All participants in the validation sample had completed the ACE-R, the predecessor of the ACE-III, plus the MMSE. All dementia patients had mild-to-moderate dementia with severity indexed by the Frontotemporal Dementia Functional Rating Scale (FTDFRS) [21], a dementia staging tool based on the assessment of everyday functional abilities and behavioural symptomatology, which has been applied to not only variants of FTD but also AD [22,23]. The FTDFRS has a raw score of 30, with higher scores denoting full functional ability. A percentage score is converted into logit scores, which aids in the spreading of patients across different severity categories and enables clinical interpretation: very mild (>4.12), mild (4.11-1.92), moderate (1.91 to −0.40), severe (−0.39 to −2.58), very severe (−2.57 to −4.99) and profound (below −4.99). All patients in the validation sample had logit scores >-0.39. First, demographic data (e.g., age, education, dementia severity and ACE-R scores) were examined in the validation sample. Then, M-ACE scores were obtained from items within the ACE-R. Scoring for orientation was modified from 0-5 to 0-4 (because of the removal of the ‘season’ item) as follows: 5 = 4, 4 = 3, 3 = 2, 2 = 1, 1 and 0 = 0. Group comparisons were made on the M-ACE and MMSE using the Kruskal-Wallis test with post hoc analyses carried out using the Mann-Whitney U test. Correlations between the M-ACE, MMSE and FTDFRS were examined using Spearman's correlation coefficient. Sensitivity, specificity, and positive and negative predictive values were calculated using discriminant analyses for the M-ACE and MMSE. Finally, the M-ACE consists of items assessing 4 main cognitive domains: orientation (to time), memory (learning and recall of the name and address), language (fluency) and visuospatial function (drawing of a clock face). Group differences were examined for percentage correct scores for each cognitive domain. For memory, the total percentage correct scores were calculated by summing up the points obtained for both learning and recall conditions. The MMSE consists of items assessing similar cognitive domains: orientation (to time and place), attention (serial 7s), memory (learning and recall of 3 items), language (written instruction, 3-stage command, sentence writing, repetition and naming) and visuospatial function (drawing of pentagons). Group differences were also examined for percentage correct scores for each of these domains on the MMSE.

## Results

Demographic data for the validation sample (n = 242) are given in table 1. Groups were matched for age (p = 0.02), years of education (p > 0.10) and years since symptom onset (p > 0.10). Disease severity, as indicated by the FTDFRS, was greater in the bvFTD than PPA group (p < 0.001). On the ACE-R, scores were lowest in the PPA group compared to the AD, bvFTD and CBS cohorts (p < 0.01), which is indicative of the large language component of this test. In addition, the AD group scored significantly lower than the bvFTD group (p < 0.01). Not surprisingly, all patients scored significantly lower than controls on the ACE-R (p < 0.001).

**Fig. 1:**
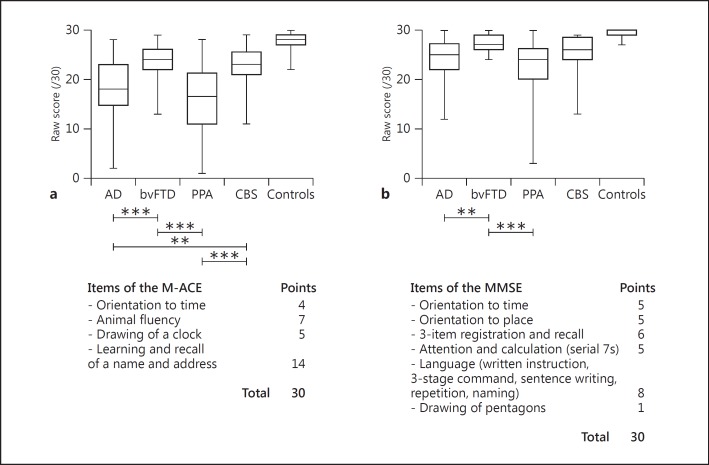
Performance on the M-ACE (**a**) and MMSE (**b**) of the patient groups and controls. Maximum and minimum scores are displayed on the box-and-whiskers graphs. All patient groups scored significantly lower than controls (p < 0.001). ** p < 0.01; *** p < 0.001.

As shown in figure 1, comparisons of the patient groups' performance on the M-ACE and MMSE indicated that all patient groups were impaired on both tests when compared to controls (p < 0.001). Intergroup comparisons for total scores revealed that the bvFTD group scored significantly higher than the AD and PPA groups on the M-ACE and MMSE (p < 0.01). On the M-ACE, the CBS group also obtained higher scores than the PPA (p < 0.001) and AD (p < 0.01) groups.

Despite very little overlap between items within the M-ACE and MMSE items, the two tests correlated strongly (r_s_ = 0.83, p < 0.001) in dementia patients. A weaker association was obtained between the M-ACE and FTDFRS, a disease staging measure (r_s_ = 0.27, p < 0.001). Reliability, assessed using Cronbach's alpha, was excellent at 0.83.

Sensitivity, specificity, positive and negative predictive values as well as likelihood ratios for the M-ACE and MMSE are shown in table 2. Sensitivity was greater on the M-ACE than MMSE at all cut-offs. A specificity of 1.0 was obtained at the lower value of 21/30 on the M-ACE (compared to MMSE, at 26/30). Two cut-offs were identified on the M-ACE. First, a cut-off of ≤25/30 has both high sensitivity and specificity values with a likelihood ratio indicating that a score of 25 is >5 times more likely to have come from a patient with dementia than without. A lower cut-off of ≤21/30 has a positive predictive value of 1.0 regardless of the prevalence, which means that a score of 21 on the M-ACE is almost certainly from a patient with dementia regardless of the clinical setting (table 3).

**Fig. 2:**
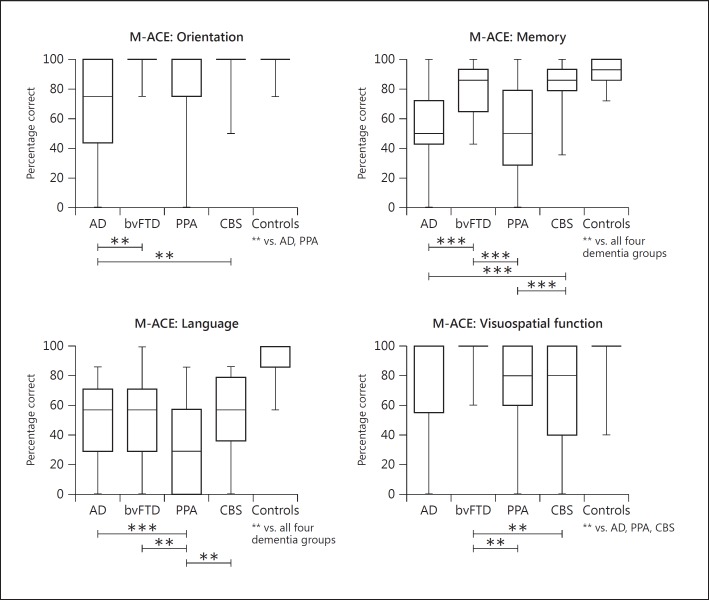
Performance on items of the M-ACE of the patient groups and controls. Maximum and minimum values are displayed on the box-and-whiskers graphs. Significant differences between patient groups are indicated with capped lines. Significant differences between patient groups and controls are indicated below the control group. ** p < 0.01; *** p < 0.001.

**Fig. 3:**
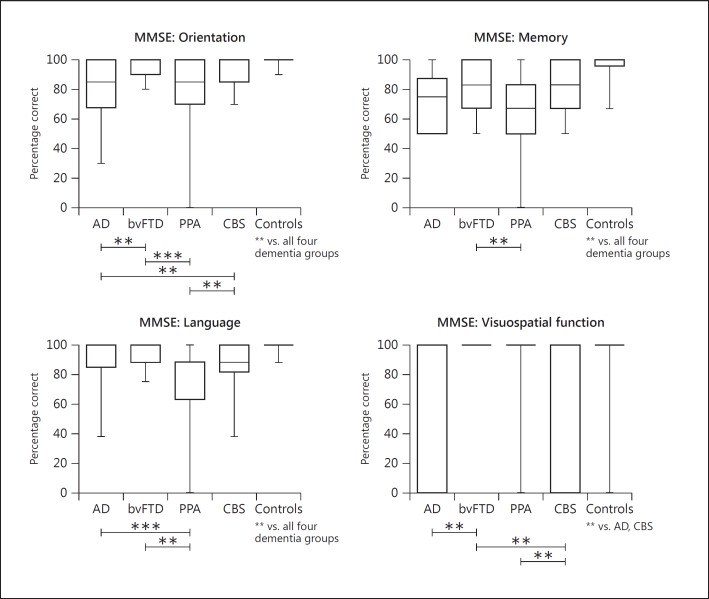
Performance on items of the MMSE of the patient groups and controls. Maximum and minimum values are displayed on the box-and-whiskers graphs. Significant differences between patient groups are indicated with capped lines. Graphs for the attention domain (serial 7s) were not included as non-significant group differences were obtained between dementia groups. Significant differences between patient groups and controls are indicated below the control group. ** p < 0.01; *** p < 0.001.

Profiles across dementia syndromes considering the main cognitive domains were examined for the M-ACE and MMSE. Percentage correct scores are displayed in figures 2 and 3. First, comparisons among the dementia cohorts for the M-ACE showed that the AD group was impaired on the orientation items compared to the bvFTD and CBS groups (p < 0.01). Memory for the name and address was significantly lower in the AD cohort than the bvFTD and CBS groups (p < 0.001). The PPA cohort also scored lower than the bvFTD and CBS groups (p < 0.001). The PPA group retrieved fewer words on the animal fluency task than all other dementia groups (p < 0.01). Finally, drawing of a clock face was worst in the CBS group compared to both FTD variants (p < 0.001). Comparisons with controls revealed that the AD and PPA groups scored significantly lower than controls on all domains (p < 0.001). The bvFTD group scored significantly lower than controls on the memory and language domains only. The CBS group scored significantly lower than controls on all domains of the M-ACE except for orientation.

Next, domain scores were examined for the MMSE. The AD and PPA groups both scored significantly lower than the bvFTD and CBS groups for the orientation items (p < 0.01). Registration and recall of 3 items was lower in the PPA cohort than the bvFTD group (p < 0.01). The PPA group also scored significantly lower than the AD (p < 0.001) and bvFTD (p < 0.01) groups for language items on the MMSE. Finally, copying of intersecting pentagons was poorer in the AD group than the bvFTD group (p < 0.01). The CBS cohort also scored lower compared to the two FTD variants (p < 0.01). No significant group differences between dementia groups were obtained for the measure of attention (serial 7s). Comparisons with controls showed that the AD and CBS group scored significantly lower on all 5 domains of the MMSE (p < 0.01). The bvFTD group scored significantly lower than controls on measures of orientation, memory and language (p < 0.01). Finally, the PPA group scored significantly lower than controls on all domains except for the copying task (p < 0.001).

## Discussion

We introduce the M-ACE, a very brief and sensitive cognitive screening tool for dementia that was derived from a data-driven scaling method. It has a maximum score of 30 with higher scores indicating better cognitive function and contains items assessing 4 main cognitive domains: orientation, memory, language and visuospatial function. Two cut-offs are suggested for clinical use or screening of patients for research. First, the higher cut-off of 25/30 has both high sensitivity and specificity and is at least 5 times more likely to have come from a patient with dementia than without. A lower cut-off of 21/30, by contrast, is almost certainly diagnostic of a dementia syndrome regardless of the prevalence rate. The M-ACE takes under 5 min to administer and, like the ACE-III, can be downloaded from http://www.neura.edu.au/frontier/research/test-downloads/.

The M-ACE has many advantages in a clinical setting. Like the MMSE, it is short and allows for the rapid assessment of patients. It contains items which are easily translated into non-English languages and does not require formal specialized training for administration or scoring. A major benefit, we feel, is the fact that the M-ACE was empirically determined and is comprised of items which are within both the ACE-III and its predecessor, the ACE-R, enabling clinicians to derive M-ACE scores from pre-existing patient data. The M-ACE was highly intercorrelated with the MMSE despite the small overlap in orientation items only. Analyses of sensitivity and specificity indicated that at all cut-offs, the M-ACE has greater sensitivity but somewhat less specificity than the MMSE. The M-ACE is, therefore, more sensitive at detecting individuals with cognitive impairment. However, impairment on test scores needs further evaluation before a dementia diagnosis is given. An additional benefit of specificity being 1.0 at the lower cut-off of 21 on the M-ACE is that this test is less likely to have ceiling effects, which are a common problem for short cognitive screening tools, and is therefore likely to be particularly useful when examining and monitoring patients in a clinical setting who have milder cognitive impairments.

Cognitive screening tools, such as the M-ACE or MMSE, do not replace the comprehensive medical, sophisticated neuropsychological and neuroimaging techniques that is required for the differential diagnosis of dementias. However, examination of total scores on the M-ACE produces somewhat distinctive cognitive profiles across not only AD and FTD variants but also CBS and replicates previous literature [24,25,26,27]. On the M-ACE, bvFTD and CBS patients both obtained higher scores than AD and PPA patients. This probably reflects the fact that a large component of the M-ACE is driven by language and verbally based (learning of name and address) items. Second, domain scores on the M-ACE showed that AD patients are impaired on tests of orientation, patients with progressive aphasias are least able to generate words on the fluency task, and drawing of a clock face is most difficult domain for the CBS cohort. While differing group profiles also emerged for the MMSE, the M-ACE was better able to differentiate dementia groups in the domains of memory and language, which are particularly important for the assessment of AD and FTD syndromes.

Symptom duration was matched across the dementia cohorts with patients exhibiting symptoms for approximately 3-4 years prior to a dementia diagnosis. On the FTDFRS, a dementia staging tool based on everyday functional abilities and behavioural symptomatology, patients in this study were rated as having mild to moderate stages of dementia. Items assessing behaviour are likely to explain why the patients with bvFTD - a syndrome characterized by striking disturbances of behaviour (disinhibition, apathy, etc.) - were rated significantly worse on the FTDFRS than the patients with PPA, a syndrome with striking language impairments particularly in the early stages of the disease. The M-ACE is a cognitive screening tool with a heavy emphasis on verbally mediated and language items. Not surprisingly, therefore, the M-ACE showed a weak association with the FTDFRS. These findings have important clinical implications. An individual with high scores on the M-ACE may in fact be in the moderate stages of dementia with significant behavioural abnormalities and functional impairments that greatly affect carer burden and may require additional medical intervention and social support services.

This study has some limitations. The M-ACE was derived using patients from specialized or tertiary hospital clinics. The applicability of the M-ACE in a community setting should be evaluated as the prevalence of dementia differs considerably and cases with mixed aetiologies are more common. Similarly, exploring the effects of age, education and sex on M-ACE scores in a population-based cohort would be valuable. Performance of patients with neurodegenerative conditions which have milder cognitive impairments should also be examined. Scores on memory items on the M-ACE were obtained from the ACE-R but the delay between learning and recall of the name and address is, however, longer in the ACE-R than the M-ACE. It is very unlikely that this will affect memory scores, particularly for patients with AD, as poor retention of new material is known to exist even after a very short interference task in AD [28,29,30]. A screening tool for executive functioning or carer-based questionnaires assessing behavioural symptomatology (e.g., disinhibition, apathy, and change in eating habits) would be useful in addition to the M-ACE when examining patients suspected of having bvFTD. Also, the utility of the M-ACE in differentiating between PPA subtypes (semantic variant, logopenic variant and non-fluent variant) is an area for further research. The M-ACE in conjunction with more specific tests of language functioning - such as the Sydney Language Battery [31] - are likely to be particularly useful in the context of assessing PPA patients in a clinical setting. Finally, while comparisons were made with the MMSE, the M-ACE should also be evaluated against other brief and freely available cognitive screening tools for dementia, such as the Montreal Cognitive Assessment [32].

The M-ACE is a brief cognitive screening tool. It has a maximum score of 30 with two cut-offs: 25 and 21. The M-ACE is highly sensitive to cognitive impairment and is less likely to have ceiling effects with a specificity of 1 being obtained at the lower cut-off. In addition, total and domain scores on the M-ACE offer somewhat distinctive profiles across FTD variant, AD and CBS syndromes, which might be useful for the differential diagnosis of dementias in a clinical setting.

## Supplementary Material

Supplementary data

## Figures and Tables

**Table 1:** Demographic, disease severity and cognitive scores in dementia groups and controls

	AD	bvFTD	PPA	CBS	Controls
Male, n (%)	38 (63)	23 (74)	82 (49]	21 (43]	78 (45]
Age, years	64.0±8.3	61.3±10.7	66.4±8.7	65.3±7.6	67.4±6.4
Education, years	12.3±2.9	11.8±2.9	11.9±3.3	11.7±3.3	12.9±2.8
Time since symptom onset, years	3.3±2.9	4.3±2.9	3,4±2.4	3.1±1.9	N/A
FTDFRS, logit score^1^	1.32±1.1	0.80±0.8^a^	1.88±1.6	1.30±0.9	N/A
ACE-R, score /100	73.2±16.1^b^	84.4±8.4	61.9±18.7^b^^c^^d^	78.6±13.3	94.6±3.3

a p < 0.01 vs. PPA.

b p < 0.01 vs, bvFTD.

c p < 0.01 vs, AD.

d p < 0.01 vs, CBS.

1 Lower scores reflect greater dementia severity.

**Table 2:** Sensitivity, specificity, positive and negative predictive values (PPV, NPV) and likelihood ratios (LR) for the M-ACE and MMSE at different cut-offs for mild-to-moderate dementia

Cut-off	M-ACE				MMSE			
	sensitivity	specificity	PPV (NPV)	LR+	sensitivity	specificity	PPV (NPV)	LR+
29	1.00	0.17	0.72 (1.00)	1.20	0.95	0.46	0.79 (0.80)	1.76
28	0.98	0.40	0.77 (0.91)	1.63	0.82	0.79	0.89 (0.67)	3.98
27	0.93	0.63	0.84 (0.80)	2.49	0.72	0.96	0.98 (0.62)	18.7
26	0.89	0.83	0.92 (0.78)	5.34	0.63	1.00	1.00 (0.56)	100
25	0.85	0.87	0.93 (0.74)	6.66	0.52	1.00	1.00 (0.50)	100
24	0.79	0.91	0.95 (0.67)	8.76	0.44	1.00	1.00 (0.46)	100
23	0.73	0.97	0.98 (0.63)	28.3	0.37	1.00	1.00 (0.43)	100
22	0.65	0.99	0.99 (0.57)	50.9	0.27	1.00	1.00 (0.40)	100
21	0.61	1.00	1.00 (0.55)	100	0.22	1.00	1.00 (0.38)	100

**Table 3:** Positive and negative predictive values (PPV, NPV] for the cut-offs of 25 and 21 on the M-ACE at different prevalence rates

Cut-off	Prevalence											
	5%		10%		20%		40%		60%		80%	
	PPV	NPV	PPV	NPV	PPV	NPV	PPV	NPV	PPV	NPV	PPV	NPV
25	0.26	0.99	0.42	0.98	0.73	0.93	0.81	0.89	0.90	0.79	0.96	0.59
21	1.00	0.97	1.00	0.96	1.00	0.91	1.00	0.79	1.00	0.63	1.00	0.39

A typical memory clinic would have a prevalence of at least 50%.
