# Supplementary material for: The Mini-Addenbrooke's Cognitive Examination: A New Assessment Tool for Dementia
Source: Dement Geriatr Cogn Disord. 2014 Sep 11;39(1-2):1–11. doi: 10.1159/000366040 (PMC4774042; doi:10.1159/000366040)
Supplement: Supplementary file 1 — Supplementary data [file dem-0039-0001-s01.docx]

**SupplementaL DATA**

**The Mini-Addenbrooke’s Cognitive Examination: A new assessment tool for dementia**

**Table 1.** Equated means of ACEIII items and subdomain scores for the entire patient group (n=117)

| **Items** | **Equated Means** |
| --- | --- |
| Orientation in Time | 0.744 |
| Orientation in Geography | 0.750 |
| 3 Item Registration | 0.932 |
| Serial Sevens | 0.576 |
| 3 Item Recall | 0.467 |
| Verbal Fluency-Letter | 0.458 |
| Verbal Fluency-Animal | 0.396 |
| Memory Anterograde | 0.777 |
| Memory Retrograde | 0.534 |
| Syntactical Comprehension | 0.789 |
| Sentence Writing | 0.543 |
| Repetition-Multisyllabic words | 0.701 |
| Repetition-All that glitters is not gold | 0.803 |
| Repetition-A stitch in time saves nine | 0.718 |
| Naming | 0.684 |
| Semantic Comprehension | 0.660 |
| Single Word Reading | 0.487 |
| Visuospatial Overlapping Infinity Loops | 0.573 |
| Visuospatial Wire Cube | 0.611 |
| Visuospatial Drawing of a Clock | 0.638 |
| Perceptual Dots | 0.821 |
| Perceptual Letters | 0.908 |
| Memory Delayed Recall | 0.326 |
| Memory Recognition | 0.744 |

Equated means are obtained by dividing the mean raw score by the maximum score for that item (e.g., a mean score of 3.97 for the item ‘Orientation in Time’ was divided by 5 to produce an equated mean of 0.74 for the entire patient cohort). Items are presented in order of the ACE-III protocol.

**Table 2.** Items ordered by difficulty and discrimination

| **Difficulty** | **Mean Score** | **Discrimination** | ***H_i_*** |
| --- | --- | --- | --- |
| **Delayed Recall** | 0.326 | Perceptual Letters | 0.542 |
| **Verbal Fluency-Animal** | 0.396 | **Memory Anterograde** | 0.518 |
| Verbal Fluency-Letters | 0.458 | **Delayed Recall** | 0.512 |
| Memory Recall | 0.467 | **Verbal Fluency-Animal** | 0.509 |
| Memory Retrograde | 0.534 | Orientation to Geography | 0.498 |
| Language Writing | 0.543 | Recognition | 0.474 |
| Serial Sevens | 0.576 | Memory Retrograde | 0.466 |
| **Visuospatial Clock** | 0.638 | Naming items | 0.451 |
| Language Comprehension | 0.660 | Memory Recall | 0.442 |
| Naming items | 0.684 | **Orientation in Time** | 0.436 |
| Repetition-A stitch in time saves nine | 0.718 | **Visuospatial Clock** | 0.419 |
| Recognition | 0.744 | Serial Sevens | 0.382 |
| **Orientation in Time** | 0.744 | Verbal Fluency-Letters | 0.367 |
| Orientation in Geography | 0.750 | Repetition-All that glitters is not gold | 0.363 |
| **Memory Anterograde** | 0.777 | Language Comprehension-Marsupial | 0.355 |
| Repetition-All that glitters is not gold | 0.803 | Language Writing | 0.319 |
| Perceptual Letters | 0.908 | Repetition-A stitch in time saves nine | 0.319 |

Mean scores range from 0-1 where lower values indicate higher difficulty. *H_i,_* provides an index of item discrimination with higher values indicate greater discriminatory ability. Items in bold represent those selected for the M-ACE.
